# Muscle, inflammation and metabolic health: is irisin the missing link?

**DOI:** 10.1590/2359-3997000000303

**Published:** 2017-12-01

**Affiliations:** 1 Universidade Federal do Rio Grande do Sul Serviço de Endocrinologia, Hospital de Clínicas de Porto Alegre Departamento de Medicina Interna Porto Alegre RS Brasil Departamento de Medicina Interna, Universidade Federal do Rio Grande do Sul (UFRGS), Serviço de Endocrinologia, Hospital de Clínicas de Porto Alegre (HCPA), Porto Alegre, RS, Brasil

In the first years of the current decade, irisin gained significant attention from the scientific community (1). A myokine, it was found to be exercise-mediated and to regulate the metabolic rate in myocytes and adipocytes, thus acting as an exercise- induced insulin sensitizer. At first, it offered a promise of a potential target of new interventions for obesity, metabolic syndrome and type 2 diabetes. As 2017 marches to the end, no real breakthroughs have happened. Nevertheless, as the interest on the effects of exercise on metabolic health gather more and more attention, research on irisin is still going strong, helping to elucidate potential hormonal pathways that will eventually lead to applicable interventions. In this issue of *Archives of Endocrinology and Metabolism,* two papers look into irisin and its potential associations with markers of metabolic status. Tabak and cols. (2) demonstrate that irisin is associated with waist circumference, waist-hip ratio, plasma LDL cholesterol levels, and inflammation markers. In the same line of reasoning, Bonfante and cols. (3) show that higher irisin levels are associated with better metabolic profile and lower risk for Type 2 diabetes mellitus in obese men. These studies stress the role of irisin as an indicator of metabolic health. A more protagonistic role for irisin in the metabolic/inflammatory cascade, in both health and disease has yet to be shown. This will be the moment when irisin will eventually become a potential therapeutic target, if ever. So far, the most important factor driving irisin and its known associations seems to be exercise. And the health impact of exercise is so noticeable and complex that irisin alone will unlikely explain the entire cohort of benefits arising from physical activity. We look forward to more investigational results on irisin, other myokynes, and the metabolic effects of exercise in the promotion of health.
